# Enhanced Cellular Doxorubicin Uptake via Delayed Exposure Following Nanosecond Pulsed Electric Field Treatment: An In Vitro Study

**DOI:** 10.3390/pharmaceutics16070851

**Published:** 2024-06-24

**Authors:** Rongwei Ma, Yubo Wang, Zhihao Wang, Shengyong Yin, Zhen Liu, Keping Yan

**Affiliations:** 1 College of Chemical and Biological Engineering, Zhejiang University, Hangzhou 310003, China; rongweima@zju.edu.cn (R.M.); 22128085@zju.edu.cn (Z.W.); kyan@zju.edu.cn (K.Y.); 2 Key Laboratory of Multi-Organ Transplantation Research, Ministry of Health, First Affiliated Hospital, School of Medicine, Zhejiang University, Hangzhou 310003, China; 12018275@zju.edu.cn (Y.W.); yinsy@zju.edu.cn (S.Y.)

**Keywords:** electroporation, electrochemotherapy, nanosecond pulsed electric field, doxorubicin

## Abstract

The combination of nanosecond Pulsed Electric Field (nsPEF) with pharmaceuticals is a pioneering therapeutic method capable of enhancing drug uptake efficacy in cells. Utilizing nsPEFs configured at 400 pulses, an electric field strength of 15 kV/cm, a pulse duration of 100 ns, and a repetition rate of 10 pulses per second (PPS), we combined the nsPEF with a low dose of doxorubicin (DOX) at 0.5 μM. Upon verifying that cells could continuously internalize DOX from the surrounding medium within 1 h post nsPEF exposure, we set the DOX exposure period to 10 min and contrasted the outcomes of varying sequences of DOX and nsPEF administration: pulsing followed by DOX, DOX followed by pulsing, and DOX applied 40 min after pulsing. Flow cytometry, CCK-8 assays, and transmission electron microscopy (TEM) were employed to examine intracellular DOX accumulation, cell viability, apoptosis, cell cycle, and ultrastructural transformations. Our findings demonstrate that exposing cells to DOX 40 min subsequent to nsPEF treatment can effectively elevate intracellular DOX levels, decrease cell viability, and inhibit the cell cycle. This research work presents a novel approach to enhance DOX uptake efficiency with moderate conditions of both DOX and nsPEF.

## 1. Introduction

In clinical practice, doxorubicin has shown considerable efficacy as a potent chemotherapeutic drug and is regarded as one of the most powerful medications approved by the Food and Drug Administration (FDA) [1]. Its ability to target rapidly dividing cells and slow disease progression has been widely recognized for several decades. The anti-tumor activity of DOX is attributed to its ability to intercalate into the DNA helix and covalently bind to proteins involved in DNA replication and transcription, thereby inhibiting the synthesis of DNA, RNA, and proteins, leading to cell death [1]. However, conventional use of DOX in chemotherapy can increase oxidative stress, contributing to cardiac toxicity, impacting cardiac tissue, and potentially causing cardiomyopathy [2]. Therefore, we tried to find an efficient release method to enhance local cell uptake at low concentrations of DOX to bolster the safety and efficacy of the treatment.

Electrochemotherapy is a treatment modality that enhances the cellular uptake of drugs by increasing cell membrane permeability through physical means, accomplished by applying pulsed electric fields locally [3]. When cells are subjected to pulsed electric fields, nanometer-sized pores appear on membranes, thereby increasing membrane permeability and enhancing drug absorption. The effect of a pulsed electric field on cell membrane permeability depends on pulse parameters such as pulse width, amplitude, pulse number [4,5,6], as well as the inherent attributes of the cells themselves, including their morphology [7] and type [8]. Nanosecond Pulsed Electric Field (nsPEF) is an emerging modality for electroporation with a similar mechanism to microsecond pulse electroporation but higher electric field strength, shorter pulse width and lower pulse energy. Beyond its influence on the cell membrane, nsPEF has been reported to induce intracellular electric stimulation, including the overload of calcium [9], phosphatidylserine translocation [10], endoplasmic reticulum stress [11], mitochondria damage [12], and other effects. The cytotoxic effect of the combination treatment of nsPEF and DOX has been proved in vitro [13,14] and in vivo [15]; however, when the exposure time of DOX is limited (10 min), nanosecond pulses with lower electric filed strength (10 kV/cm) showed poor synergic effect [14].

During the combination treatment of pulsed electric field and anticancer drugs, the administration of drugs usually before or right after pulse delivery, for the lifetime of the pore, was thought to be several minutes post pulsed electric field treatment [4,16]. When the pores were on the membrane enclosure, the lipophobic agents could not enter cells. However, doxorubicin has the ability to penetrate cells due to its lipophilic characteristics and DNA intercalating/ binding properties [1]. It is reported that during the combination of microsecond pulse and DOX, the cell uptake of DOX still increased 1 h post pulse delivery [17]. Therefore, the timing of DOX administration should not be limited by the window time of pore existing. With the time of the DOX exposure limited, it is essential to compare the different timing of DOX administration during the combination treatment of nsPEF and DOX, especially a delayed exposure of DOX following nsPEF treatment, which has not been mentioned in a previous study. In this paper, the cellular uptake of DOX was measured 1 h post nsPEF treatment. Then, with the exposure time limited to 10 min, the impact of DOX administration timing on the drug uptake, cell activity, apoptosis, cell cycle, and ultrastructural changes with DOX prior pulse, DOX post pulse and DOX 40 min post pulse was compared.

## 2. Materials and Methods

### 2.1. Cell Culture

Human hepatocellular carcinoma (HCC) cell line Hep3B was used in this study. Cells were cultured in MEM, supplemented with 10% FBS containing 5% CO_2_ at 37 °C.

### 2.2. Pulse Generator and nsPEF Application

**Figure 1:**
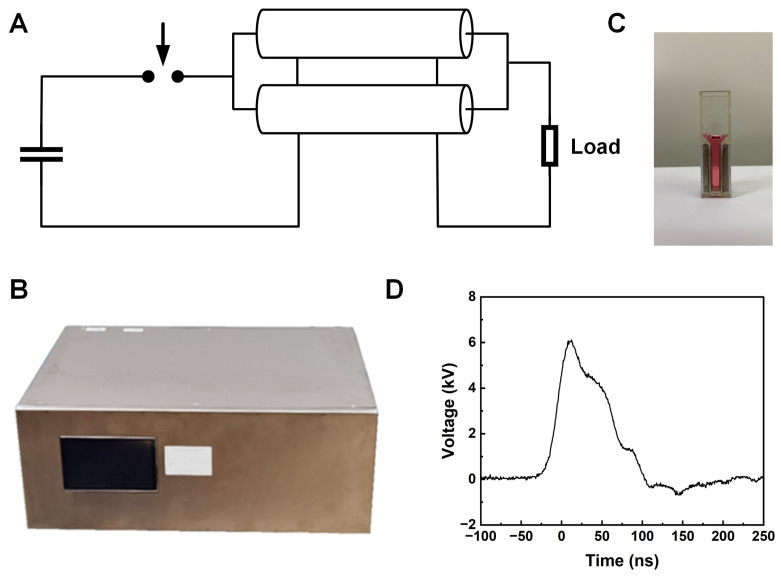
The schematic diagram of the experimental setup for nsPEF for Hep3B cells. (**A**) the circuit of nsPEF generator (**B**) demonstration of nsPEFs generator (**C**) cuvettes (**D**) the waveform of the pulse delivered to cuvettes.

The homemade pulse generator adapted a transmission line transformer with an output impedance of 25 Ω (Figure 1A,B), which matches the impedance of the cuvette with a 4 mm electrode gap (Biosmith, Vandergrift, PA, USA) (Figure 1C) [18]. The generator consistently delivered pulses with an amplitude of 6 kV (Figure 1D) and a repetition rate of 10 pulses per second (PPS) to the cuvette. Under these conditions, the average electric field strength was 15 kV/cm.

Hep3B cells were harvested with trypsin (Gibco-Invitrogen, Carlsbad, CA, USA), and then resuspended in MEM containing 10% FBS to a concentration of 2.0 × 10^6^ cells/mL. For each nsPEF application, 800 μL of cell suspension was placed into the cuvette and treated with a same electric field strength of 15 kV/cm and a pulse repetition rate of 10 PPS. To identify the most appropriate pulse number, the pulse number was adjusted from 200 to 1000. Subsequent to this optimization, the pulse number that yielded the best results was selected and thereafter maintained constant for all subsequent experiments.

### 2.3. Doxorubicin Treatment

For the doxorubicin treatment, cells were detached with trypsin and then dispensed into 96-well plates. Doxorubicin was dissolved in dimethyl sulfoxide (DMSO) and then diluted in complete medium to prepare a series of gradient concentrations of solutions. Cells were incubated with DOX (0.0625, 0.125, 0.25, 0.5, 1, 2, 4, 8 μM) for 24 h and 48 h, after which cell viability was assessed using CCK-8 assay.

### 2.4. Microscopy

Propidium iodide (PI) was used as the fluorescent marker of membrane permeabilization. Hep3B cells were treated with 400 pulses and incubated in complete culture medium for 10, 20, 40, and 60 min at room temperature. Subsequently, the cells were washed twice with PBS and stained with 100 μL/mL of PI. The cell suspension was then pipetted into glass-bottom dishes for observation under an inverted fluorescence microscope (FIM). The PI-positive rate was defined as the ratio of PI-positive cells to the total number of cells.

### 2.5. Quantitation of Doxorubicin Uptake and Efflux

To analyze the DOX uptake, flow cytometry was employed. The intracellular doxorubicin fluorescent intensity (reflecting accumulated fluorescence) was quantified by flow cytometry (FACS LSRII, BD Bioscience, San Diego, CA, USA) with an excitation wavelength of 488 nm and an emission wavelength exceeding 560 nm [19].

To obtain the temporal changes in cell uptake in 1 h post nsPFE, cells were detached with trypsin and then subjected to a 400-pulse nsPEF treatment at 15 kV/cm and a pulse repetition rate of 10 PPS. The treatment medium contained 0.5 μM of DOX and a complete culture medium. Cells were kept in a medium for intervals of 10, 20, 40, and 60 min, then centrifuged and washed twice with PBS. Another group of cells was incubated only in 0.5 μM of DOX for the same time intervals and washed with PBS twice before flow cytometry measurement. Cells subjected to neither DOX nor nsPEF were set as the control group. Suppose the mean fluorescent intensity of each group is I, the mean fluorescent intensity of cells incubated in DOX for 1 h is $I_{DOX\_1h}$, and the mean fluorescent intensity of cells treated by neither nsPEF nor DOX is $I_{control}$. The relative fluorescent intensity of cells in each group is calculated as follows:

(1) $$Relative\;fluorescent\;intensity_{1}=\frac{I-I_{control}}{I_{DOX\_1h}-I_{control}}\times 100\%$$

**Figure 2:**
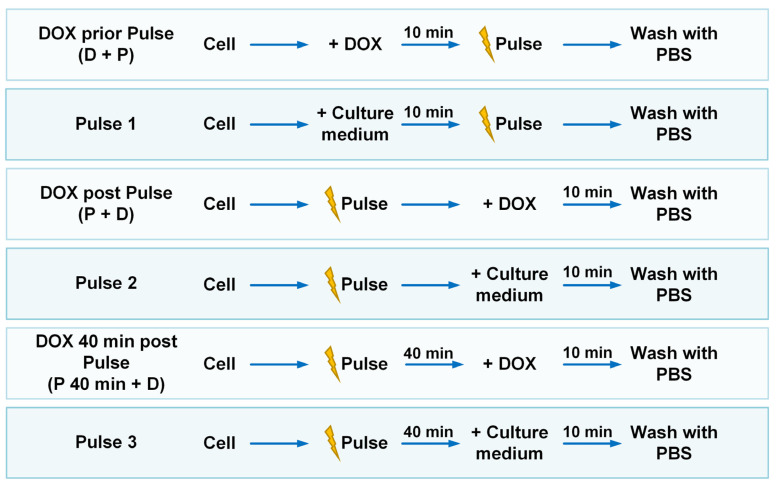
The experimental procedures of nsPEF applied with DOX or culture medium at different administration timings.

Cells with varied DOX administration timings (Figure 2) were incubated in PBS at 37 °C after nsPEF and DOX application. The DOX signal was detected after 10 min and 40 min of incubation. Another group of cells was treated only with 0.5 μM DOX for 10 min, washed twice with PBS, and then incubated in PBS for 10 and 40 min. Suppose the mean fluorescent intensity of each group is I, the mean fluorescent intensity of cells treated with DOX for 10 min followed by a PBS incubation for 10 min is $I_{PBS\_10\;min}$, and the mean fluorescent intensity of cells treated with neither nsPEF nor DOX is $I_{control}$. The relative fluorescent intensity of cells in each group is calculated as follows:

(2) $$Relative\;fluorescent\;intensity_{2}=\frac{I-I_{control}}{I_{PBS\_10\;min}-I_{control}}\times 100\%$$

### 2.6. Combination Treatment of Doxorubicin and nsPEF with Different Timing of DOX Administration

For combined treatment with nsPEF and doxorubicin, the concentration of DOX was 0.5 μM and the pulse number was set to 400 pulses. The study compared the effects of three distinct timing strategies for DOX administration: DOX administered before the pulse (DOX prior pulse, D + P), DOX given after the pulse (DOX post pulse, P + D), and DOX administered 40 min post pulse (DOX 40 min post pulse, P 40 min + D). Across all three groups, the duration of DOX incubation was standardized to 10 min, with variation lying in the sequence and interval between DOX administration and pulse application. The detailed procedure is as follows. (1) In the first regimen, cells are first incubated in DOX and then subjected to pulse treatment in a medium containing DOX. Afterward, they are immediately centrifuged and washed twice with PBS. (2) In the second regimen, cells are subjected to pulse treatment in a pulsing medium without DOX, immediately followed by a 10 min incubation with DOX, and then centrifuged and washed twice with PBS. (3) In the third regimen, cells are subjected to pulse treatment in a DOX-free medium, after which there is a 40 min interval before DOX is added for a 10 min incubation, followed by being centrifuged and washed twice with PBS. In addition to the combination treatment of nsPEF and DOX, identical procedures are carried out where the medium containing DOX is replaced with a DOX-free culture medium to evaluate the effects of applying nsPEF alone. All these procedures are performed at room temperature. The cell viability, DOX uptake, apoptosis, cell cycle and ultrastructure of cells are analyzed. The synergistic quotient (SQ) is calculated by dividing the effect of the combination treatment by the sum of the individual effects of the two treatments [20]. A value exceeding 1.0 suggests synergism for the measured response.

### 2.7. Cell Viability

Cell viability was determined by the CCK-8 (Dojindo, Kumamoto, Japan) assay. Cells were seeded into 96-well plates and incubated with 10 μL of CCK-8 solution at 37 °C. Each sample was replicated six times. After 1 h of incubation, the optical density (OD) was measured at 450 nm using a spectrophotometer (ELx800; BioTek Instruments, Inc., Vermont, VT, USA). The relative survival rate was calculated by dividing the OD values of the experimental samples by those of the control group.

### 2.8. Apoptosis Assay

Cells were stained using the Annexin V-FITC/PI Apoptosis Detection Kit (Dojindo, Kumamoto, Japan). According to the manufacturer’s instructions, cells were suspended in 200 μL of a binding buffer. Subsequently, 10 μL of Annexin V-FITC and 10 μL of propidium iodide (PI) staining solution were gently mixed into the cell suspension. The mixture was then kept in the dark at 25 °C for 15 min. In total, 800 μL of the binding buffer was added to terminate the staining reaction. Finally, the stained cells were examined via flow cytometry analysis.

### 2.9. Cell Cycle Analysis

The cells were digested and then fixed in 75% pre-cooled ethanol at 4 °C for 1 day. After the fixation process, the cells were washed and resuspended with the Cell Cycle Staining Kit (Multi Sciences, Hangzhou, China) at room temperature. Subsequently, the cell cycle distribution was determined using flow cytometry.

### 2.10. Transmission Electron Microscope

Cells were fixed with 2.5% glutaraldehyde in phosphate buffer over 4 h, after which they were thoroughly washed with PBS three times. The samples were then post fixed with 1% OsO4 in phosphate buffer for a period of 1–2 h, followed by another round of three PBS washes. Subsequently, the samples were dehydrated by a graded series of ethanol solutions (30%, 50%, 70%, 80%), followed by acetone solutions (90%, 95%), and finally pure acetone. The dehydrated specimens were then embedded in Spurr resin. Thin sections were cut using a Leica EM UC7 (Wetzlar and Mannheim, Germany), and stained by uranyl acetate and alkaline lead citrate. Finally, the stained sections were visualized with a Hitachi Model H-7650 transmission electron microscope (TEM) (Tokyo, Japan).

### 2.11. Statistical Analysis

The raw data were normalized using Microsoft Excel 2021. GraphPad Prism 8.0 was utilized to generate graphs, and FlowJo V10 was used to analyze Flow cytometry data. Student’s *t*-test and one-way ANOVA were performed to evaluate variance, with significance set at *p* < 0.05. All experiments were repeated three times.

## 3. Results

### 3.1. Determining the Concentration of Doxorubicin and Pulse Parameters

**Figure 3:**
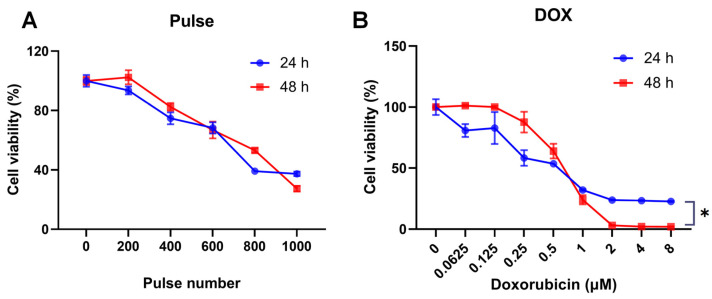
(**A**) Cell viability decreased as the number of pulses increased. Hep3B cells were treated with 0–1000 pulses of nsPEF at 15 kV/cm. Cell survival rates were assessed at 24 and 48 h post treatment with the indicated number of pulses. (**B**) Cell viability decreased as the concentration of DOX increased. Hep3B cells were exposed to 0–8 μM DOX for 24 and 48 h. *: *p* < 0.05.

To determine the appropriate number of pulses, we delivered 200 to 1000 pulses to the cuvette and compared cell viability at 24 h and 48 h intervals, as shown in Figure 3A. As the number of pulses increased, cell viability decreased, reaching a point where about half of the cells died when the number of pulses exceeded 800. The survival trends were similar at 24 h and 48 h. The nsPEF pulses exert cumulative effects on cells, ultimately influencing cell proliferation and survival [21]. To ensure a sufficiently pronounced effect of nsPEF and avoid excessive damage to cells at the same time, the pulse number was set as 400 in the following experiments.

We evaluated the toxicity of DOX on Hep3B cells. The IC50 values for a 24 h and 48 h exposure to DOX were 0.541 ± 0.049 and 0.621 ± 0.014, respectively. As shown in Figure 3B, at concentrations below 0.5 μM, the cytotoxic effect was found to be weaker at 48 h compared to 24 h; conversely, at concentrations above 0.5 μM, it became more pronounced after 48 h of DOX exposure. This indicates that the differential effect of DOX dosage become more evident after a 48 h treatment period. Consequently, for subsequent experiments involving DOX in combination with nsPEF, we chose a concentration of 0.5 μM to observe its cytotoxic effect.

### 3.2. The Intercellular Uptake of DOX Increased within 1 h Post nsPEF Exposure

**Figure 4:**
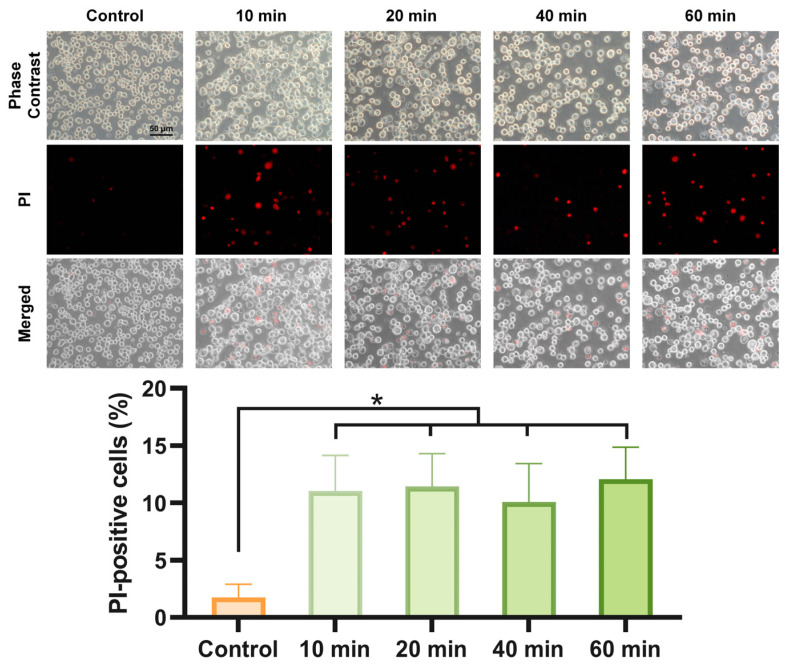
NsPEF treatment increased cell permeability, with the fraction of PI-positive cells remaining stable within 1 h post treatment. Cell permeability within 1 h after 400 pulses of nsPEF was evaluated by PI uptake. Phase contrast and PI fluorescence images were taken to analyze the number of PI-positive cells and the total cell count. *: *p* < 0.05.

PI was utilized to assess membrane permeability 1 h post pulse. As depicted in Figure 4, PI uptake was evident following nsPEF treatment. Compared to the control group, the number of PI-positive cells increased significantly after 400 pulses of nsPEF treatment (*p* < 0.05). However, within the 1 h period following nsPEF treatment, no significant differences in the PI-positive rate were observed at the various time points. The PI-positive rate varied from 7.1% to 15.6% among all groups treated with 400 pulses of nsPEF, with an average PI-positive rate of 11.2% ± 2.7%.

**Figure 5:**
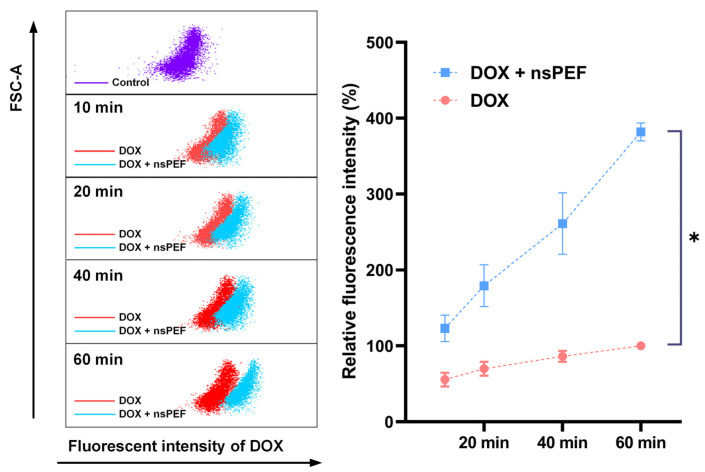
NsPEF consistently enhanced intracellular DOX uptake within 1 h after treatment. For the DOX + nsPEF group, Hep3B cells were subjected to 400 pulses of nsPEF and then incubated in a pulsing medium containing a complete culture medium and 0.5 µM of DOX for 10–60 min. For the DOX group, cells were incubated directly in DOX for 10–60 min. The control group consisted of cells treated with neither nsPEF nor DOX. Flow cytometry was used to measure the intracellular fluorescence intensity of DOX. The relative fluorescence intensity of each group was calculated as the ratio of the difference between the mean fluorescence intensity of each group and the control group to the difference between the intensity of cells in the DOX group at 60 min $\left(I_{DOX\_60min}\right)\;$and the control group. *: *p* < 0.05.

To quantify the uptake of doxorubicin by cells post nsPEF treatment, we utilized flow cytometry to measure the fluorescence intensity of doxorubicin. As shown in Figure 5, upon exposure to doxorubicin, regardless of whether the cells had undergone nsPEF treatment, the intracellular concentration of doxorubicin progressively increased over time. Particularly, cells treated with nsPEF followed by a 10 min incubation with doxorubicin displayed a higher intracellular fluorescence intensity compared to the cells incubated with doxorubicin alone for 1 h.

Taking into consideration the FSC (forward scatter) signals, which vary according to cell size [22], we found that the FSC signals in groups subjected to both nsPEF and DOX were all higher than those in the group treated solely with DOX, and increased in 1 h post nsPEF, indicating an increasing volume of cells caused by nsPEF.

### 3.3. Comparing the Effects of Three Different Timing Strategies for Drug Administration

**Figure 6:**
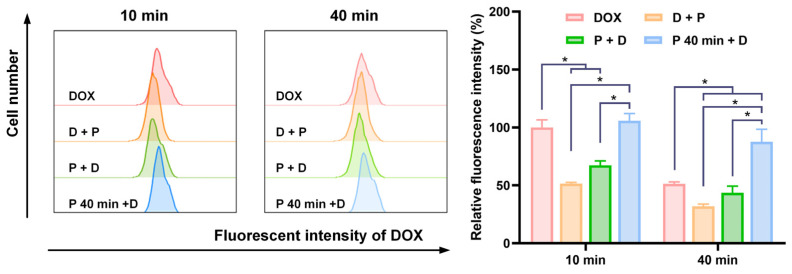
The introduction of doxorubicin 40 min post pulse led to increased intracellular doxorubicin uptake. Cells were treated with DOX or a combination of nsPEF and DOX. In the DOX group, cells were incubated with DOX for 10 min and washed twice with PBS. In the D + P, P + D, and P 40 min + D groups, DOX was administered at different timings relative to nsPEF, with a 10 min incubation period identical to the DOX group. Cells were washed with PBS twice after treatment. The control group was sham-exposed to nsPEF and DOX, followed by PBS washing. After PBS washing, cells from all groups were incubated in PBS at 37 °C for 10 to 40 min. Intracellular DOX concentration was measured using flow cytometry. The relative fluorescent intensity of each group was calculated as the ratio of the difference between the mean fluorescent intensity of each group and the control group to the difference between the intensity of cells in the DOX group at 10 min $\left(I_{DOX\_10min}\right)$ and the control group. *: *p* < 0.05.

A high intracellular concentration of DOX can be achieved by incubating with DOX for merely 10 min after nsPEF exposure. Thus, we confined the DOX incubation period to 10 min and assessed the impact of varying the sequence of DOX and nsPEF application. The DOX administration timings were Dox prior pulse (D + P), DOX post pulse (P + D) and DOX 40 min post pulse (P 40 min + D), which were shown in Figure 2. However, as shown in Figure 6, we found that when there was no time interval with the application of DOX and nsPEF, the intracellular DOX concentrations were lower compared to those in cells not subjected to nsPEF treatment. Only when DOX was applied 40 min after nsPEF treatment, a higher cellular concentration of DOX could be achieved. At 40 min post DOX administration, the intracellular DOX levels decreased in all groups compared to those measured at 10 min, and the DOX concentration in the P 40 min + D group remained the highest.

**Figure 7:**
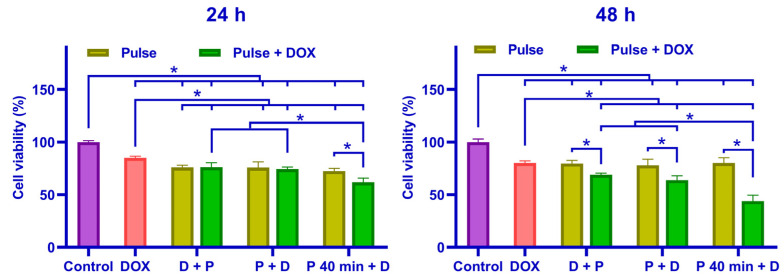
Cell viability was assessed at 24 h and 48 h post treatment. The pulse + DOX group included different DOX administration timings (0.5 μM): D + P, P + D, and P 40 min + D. The pulse group involved pulsing applied with different culture medium timings corresponding to the pulse + DOX group. Cells in the DOX group were treated with 0.5 μM DOX for 10 min and washed with PBS twice. The control group consisted of cells sham-exposed to nsPEF and DOX. * *p* < 0.05.

The cell viability of each group is illustrated in Figure 7, with the SQ value shown in Table 1. At 24 h post treatment, cell viability significantly decreased in groups subjected to the combined treatment of nsPEF and DOX compared to DOX alone, and only the P 40 min + D group exhibited decreased cell viability compared to nsPEF application alone. At 48 h post treatment, all groups under the combination treatment of nsPEF and DOX demonstrated reduced cell viability compared to the application of nsPEF or DOX alone. Considering the timing of drug administration, regardless of whether it was 24 or 48 h post treatment, the P 40 min + D group consistently showed a significant decrease in cell viability, and when there was no time interval between nsPEF and DOX application, the sequence of applying DOX and nsPEF did not significantly affect cell survival rates. When cells were subjected only to nsPEF, there was no significant difference in cell viability when the culture medium was added at different time points. The SQ value at 48 h exceeded that at 24 h across all groups, indicating a more substantial combined effect of doxorubicin and nsPEF at the later time point. Only the group with a 40 min interval between DOX and nsPEF application exhibited an SQ value exceeding 1, which indicated a synergistic effect.

**Figure 8:**
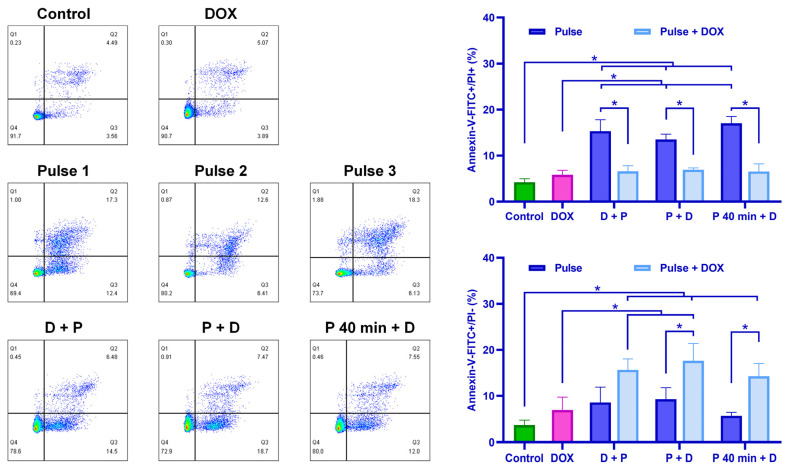
The addition of DOX resulted in decreased necrosis and increased apoptosis. Cell death in Hep3B cells was evaluated 30 min post treatment with DOX, nsPEF, and their combination using flow cytometry. The percentage of necrotic (or late apoptotic) and early apoptotic cells in each group was quantified based on the flow cytometry results. *: *p* < 0.05.

The apoptotic status of cells under three different timing strategies for drug administration was analyzed with flow cytometry. As shown in Figure 8, the timing of culture medium addition had no significant impact on cell apoptosis when nsPEF was applied alone. Similarly, when nsPEF was combined with DOX, the timing of DOX administration did not significantly affect cell apoptosis. However, after nsPEF treatment alone, the proportion of late apoptotic/necrotic cells (annexin V-positive, PI-positive) increased significantly compared to the combination treatment of nsPEF and DOX, DOX treatment alone, and the control group. When cells were under combined treatment of nsPEF and DOX, the early apoptosis rate (annexin V-positive, PI-negative) in all groups was higher than that in the control group. Specifically, the early apoptosis rates in the P + D and P 40 min + D groups were significantly higher than with nsPEF alone (*p* < 0.05), and the apoptosis rates in the D + P and P + D groups were significantly higher than with DOX alone.

**Figure 9:**
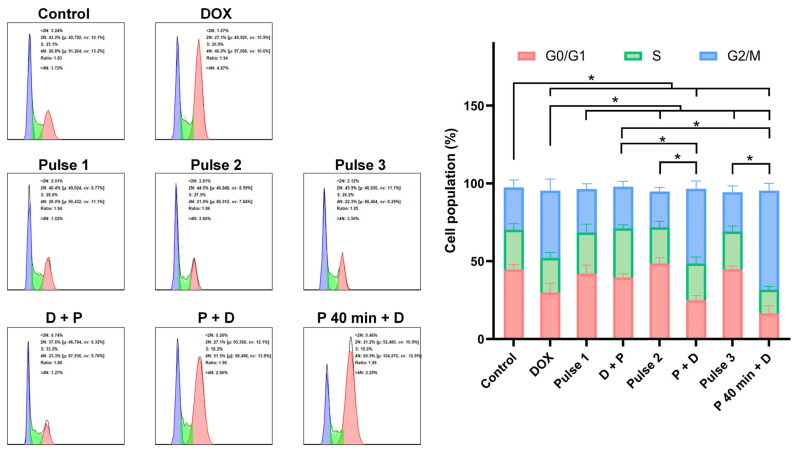
Adding DOX 40 min post nsPEF effectively inhibits the cell cycle. Cell cycle distribution of cells treated with DOX, nsPEF, and their combination at different administration timings was determined using flow cytometry at 48 h post treatment. The percentages of cells in each phase of the cell cycle were presented in a histogram. *: *p* < 0.05.

The impact of the drug administration timing is particularly significant on the cell cycle. As shown in Figure 9, we found that nsPEF did not have a significant impact on the cell cycle, regardless of the time point at which the culture medium was added, whereas 0.5 μM of DOX already exhibits inhibitory effects on the cell cycle. When DOX was applied before nsPEF treatment, its impact on the cell cycle was negligible. In P + D group, its inhibitory effect on the cell cycle was essentially comparable to treating cells with DOX alone. The most significant inhibition of the cell cycle occurred when DOX was administered 40 min post nsPEF, which aligns with cell survival outcomes.

### 3.4. The Changes in Cellular Ultrastructure

**Figure 10:**
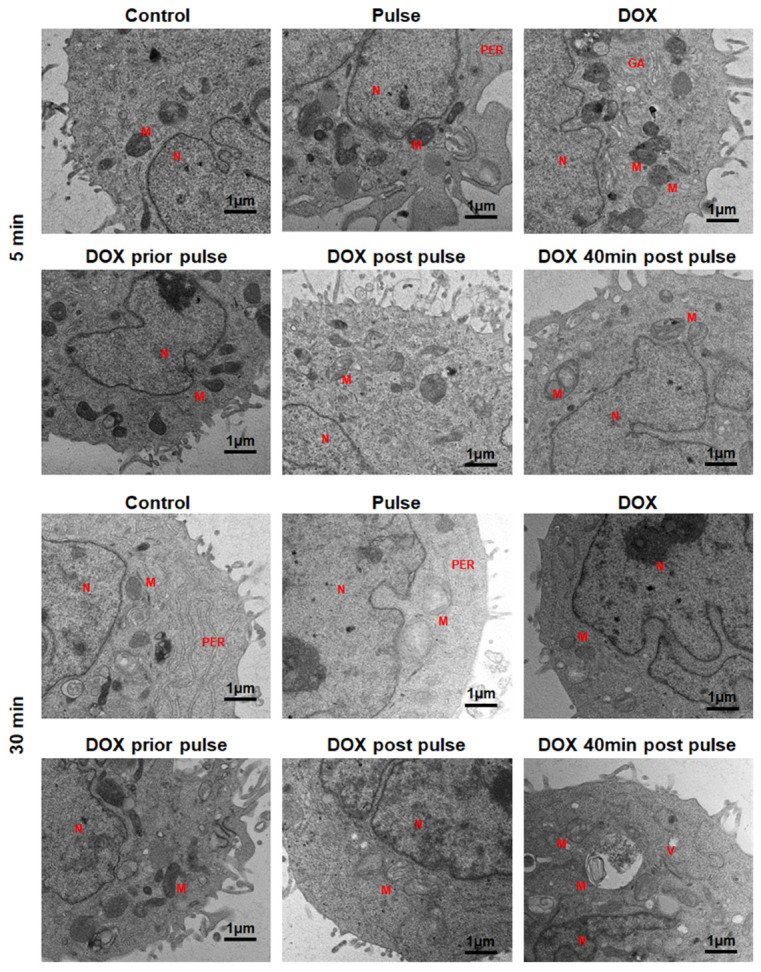
The cellular morphology changes 5 min and 30 min post the combination application of nsPEF and DOX. Abbreviations: N-nucleus; M-mitochondria; GA-Golgi apparatus; V-vesicle; PER-rough endoplasmic reticulum.

TEM observation showed alterations in the ultrastructural morphology of Hep3b after nsPEF and DOX treatments (Figure 10). In the untreated control group, nuclei with regular shapes and invaginated nuclear envelopes, condensed mitochondria, rough endoplasmic reticulum, and intact cell membrane with microvilli could be observed. Five minutes post nsPEF treatment alone, the microvilli on the cell membrane disappeared, and the cell membrane wrinkled. After 30 min, translucent mitochondria with a few cristae were observed, and the microvilli on cell membrane disappeared. The elimination of cellular microvilli can be attributed to cell swelling, and once cells recover from this swelling, microvilli tend to reappear [23,24].

When nsPEF was combined with DOX without any time interval between them (D + P and P + D), 5 min post joint treatment, the microvilli on the cell membrane remained observable, and the cell morphology did not exhibit any significant deviation from that of the control group. Thirty minutes post treatment, cells exhibited mitochondrial swelling, uneven intracellular electron density, and cytoplasmic vacuolization characterized by the presence of small vacuoles. The microvilli on the cell membrane disappeared in the P + D group, whereas in the D + P group, the microvilli still remained. When pulses and DOX were applied with a 40 min delay, mitochondrial swelling and the disappearance of microvilli were observed just 5 min post combined treatment, with cell morphology aligning with that observed 30 min post nsPEF. After 30 min, cells showed abundant cytoplasmic vacuoles, swelling mitochondrial and the absence of microvilli.

DOX is a lipophilic compound that incorporates an amine group, which can trigger temporary vacuolization [25]. Hence, the size and quantity of vacuoles within cells 30 min post treatment can serve as an indicator of DOX’s efficacy in entering the cells. The group treated with DOX 40 min after the pulse exhibited the most prominent intracellular vacuoles, indicating the highest intracellular concentration of DOX, which aligns with the results from the flow cytometry measurement of the intracellular DOX concentration.

## 4. Discussion

In this study, we found that PI uptake by cells remained at a low level within 1 h after 400 pulses of nsPEF and did not change over time, whereas DOX uptake by cells continued to increase, accompanied by cell volume increase. The cell swelling induced by nsPEF is attributed to the differential permeability of the pores on the cell membrane to substances of different sizes. When small intracellular and extracellular solutes can pass through the cell membrane to reach osmotic equilibrium, larger intracellular solutes cannot pass through the cell membrane, and cause an increased osmotic pressure inside the cell, resulting in water influx and cell swelling [4,26]. The difference in the PI and DOX uptake capacity of cells is due to the difference in membrane permeability to these substances. PI can only enter and exit cells through pores in the cell membrane, while DOX, a lipophilic drug, has a strong ability to pass through the cell membrane. Within 1 h after 400 pulses, the PI positive rate was about 10% and did not change over time, indicating that 400 pulses of nsPEF had an effect on permeability for at least 1 h. When the cells were incubated in DOX, the penetration of solutes toward equilibrium could cause continuous swelling 1–2 h post pulse [27,28]. The penetration ability of DOX maintained a high osmotic pressure in cells through continuous entry, resulting in the accumulation of DOX and cell swelling.

The removal of cells from the DOX environment resulted in a decrease in intracellular DOX concentration. This decrease could be attributed to the free diffusion of DOX and the cellular efflux of the drug. The group treated with DOX alone exhibited the greatest decrease in intracellular DOX concentration from 10 to 40 min post treatment. Kulbacka et al. reported a decreased expression of P-glycoprotein (P-gp) when gastric and colon cells were treated with 5 square electric pulses of 1300 V/cm and 50 μs pulses in combination with 1.7 μM DOX [29]. P-gp is a transmembrane efflux pump that actively transports substances out of the cell against their concentration gradients using ATP. The combination of nsPEF and DOX may reduce drug efflux by lowering the expression of P-gp protein.

We found that when nsPEF was combined with DOX, the 40 min interval did not cause significant changes in apoptosis, consistent with the finding that PI uptake by cells remained relatively stable within 1 h after the pulse. The apoptosis of cells at different times after the pulse may be related to pulse intensity. Beebe [30] et al. treated Jurkat cells with three 60 ns pulses of 60 kV/cm, observing that apoptotic cells decreased from 82.2% ± 1.3% to 70.0% ± 2.8% from 5 min to 30 min post pulse, while necrotic cells increased from 12.8% ± 1.4% to 26.8% ± 3.7%. Similarly, Kasprzycka [22] et al. treated Leydig TM3 mouse testicular cells with 80 pulses of 14 kV/cm nanosecond pulses, finding that apoptosis remained stable 30 min after the pulses, but decreased while necrosis increased at 60 min. These results suggest that some apoptotic cells transition to necrosis over time post nsPEF, with the rate of transformation potentially related to pulse intensity. Pakhomova [27] et al. demonstrated that adding sucrose to the medium could inhibit cell swelling, maintaining more cells in the apoptotic and living state rather than necrosis. They proposed that the introduction of sucrose slowed down rapid cell necrosis caused by nsPEF. In this study, the apoptosis state of cells remained relatively stable 40 min after pulses, indicating a slow transformation from apoptosis to necrosis under the pulse conditions we used. Additionally, we found that combining nsPEF with DOX reduced the proportion of the late apoptotic/necrotic cells caused by nsPEF and increased the proportion of apoptotic cells. This effect is possibly due to DOX having a protective effect similar to sucrose. When cells were incubated in DOX, extracellular DOX increased the extracellular osmotic pressure. When cells left the DOX environment, intracellular DOX concentration decreased through free diffusion. Both processes inhibited cell swelling and slowed the transition of cells from apoptosis to necrosis. Concurrently, DOX promotes apoptosis by causing oxidative stress and mitochondrial damage [31]. Therefore, the combination of nsPEF and DOX resulted in an increased proportion of early apoptotic cells and a decreased proportion of late apoptotic/necrotic cells.

In the observation of cell ultrastructure, mitochondrial swelling was noted 30 min after nsPEF treatment, which was not observed 5 min post pulse. The mechanisms of swelling are not clear yet. It is reported that calcium overload and high levels of reactive oxygen species (ROS) lead to alterations in mitochondrial membrane permeability, resulting in the opening of non-selective and high-conductance permeability transition pores (PTPs) in the inner mitochondrial membrane (IMM) [32,33]. Ions and mitochondrial membrane potential (ΔΨm) are key factors regulating matrix swelling [34]. Studies have shown that nsPEF induces ROS production [35,36] and ΔΨm decreasing [12,37]. Beebe et al. observed more pronounced dissipation of ΔΨm at 30 min post nsPEF treatment compared to 10 min [12]. Frandsen et al. employed 8 pulses of 99 μs and 6.6 kV/cm for cell electroporation, and noted a decline in intracellular ATP levels 1 h after the pulse [38]. ΔΨm serves as the driving force for ATP synthesis in mitochondria, and disruptions in membrane potential homeostasis may impair ATP synthesis [39]. Therefore, we hypothesized that mitochondrial function exhibits more pronounced impairment 40 min post pulse compared to immediately post pulse in our experiment. Furthermore, doxorubicin specifically binds to cardiolipin, abundant in the inner mitochondrial membrane, initiating ROS production and causing substantial damage to the mitochondrial structure, ultimately leading to cell apoptosis [40,41]. Hence, the synergy between nsPEF and DOX on mitochondria might be enhanced, especially when doxorubicin is administered 40 min post nsPEF treatment.

The impact of the timing of drug administration on the cytotoxic effects of low-dose drugs combined with nsPEF was investigated in this paper. We found that when the exposure time of DOX is limited to 10 min, a delayed DOX administration following nsPEF resulted in better cytotoxic effects compared to administering DOX before or immediately after nsPEF application. Enomoto et al. used a low concentration of DOX (0.03 μM) in combination with nsPEF, with DOX administered 24 h after nsPEF exposure, resulting in lower cell survival rates compared to administering DOX before nsPEF [13]. Zhang et al. found that when low concentrations (100 ng/mL) of paclitaxel were combined with nanosecond pulses, a synergy effect could be observed when the drug was administered after the pulses or when the drug was present during and after the pulses. When the drug was exposed only during the pulse period, there was no synergistic effect, which indicates that large amounts of the drug did not enter the cells during the pulse period [42]. These studies collectively highlight the importance of the presence of drugs in the cellular environment following nsPEF exposure.

## 5. Conclusions

In this paper, we proposed to improve DOX delivery efficiency through delayed DOX administration following nsPEF treatment for the first time. We found that nsPEF treatment significantly enhances the cellular uptake of DOX post pulse and exhibits a time-dependent effect. When the exposure time to DOX is limited, delayed DOX administration after nsPEF application results in higher drug uptake, cell cycle inhibition, and cytotoxicity compared to pre-pulse or immediate post pulse DOX administration. Our findings suggest that adjusting the timing of DOX administration can enhance the synergistic efficiency of nsPEF combined with the low-dose, short-exposure of DOX, providing valuable insights for practical applications.

## Figures and Tables

**Table 1:** Synergistic Quotient (SQ) for Hep3b Survival.

	D + P	P + D	P 40 min + D
24 h	0.61	0.65	0.90
48 h	0.78	0.82	1.40

## Data Availability

The data presented in the study are available on the request from the corresponding author.
